# Drug dosage in isolated limb perfusion: evaluation of a limb volume model for extremity volume calculation

**DOI:** 10.1186/1477-7819-12-81

**Published:** 2014-04-01

**Authors:** Lars Erik Podleska, Thorsten Poeppel, Michael Herbrik, Lisa Dahlkamp, Florian Grabellus, Georg Taeger

**Affiliations:** 1 Department of Trauma Surgery and Musculoskeletal Surgical Oncology, University Hospital of Essen and Sarcoma Center at the West German Cancer Center (WTZ), University of Duisburg-Essen, Hufelandstr. 55, D-45122 Essen, Germany; 2 Department of Nuclear Medicine, University Hospital of Essen, University of Duisburg-Essen, Essen, Germany; 3 Department of Diagnostic and Interventional Radiology and Neuroradiology, University Hospital of Essen, University of Duisburg-Essen, Essen, Germany; 4 Institute of Pathology and Neuropathology, University Hospital of Essen and Sarcoma Center at the West German Cancer Center (WTZ), University of Duisburg-Essen, Essen, Germany; 5 Department of Musculoskeletal Surgical Oncology, University Hospital of Essen and Sarcoma Center at the West German Cancer Center (WTZ), University of Duisburg-Essen, Essen, Germany

**Keywords:** TNF-alpha, Melphalan, Volumetry, Sarcoma, Regional perfusion, Isolated limb perfusion, Drug dosage

## Abstract

**Background:**

Exact drug dosing in isolated limb perfusion (ILP) and infusion (ILI) is essential. We developed and evaluated a model for calculating the volume of extremities and compared this model with body weight- and height-dependent parameters.

**Methods:**

The extremity was modeled by a row of coupled truncated cones. The sizes of the truncated cone bases were derived from the circumference measurements of the extremity at predefined levels (5 cm). The resulting volumes were added. This extremity volume model was correlated to the computed tomography (CT) volume data of the extremity (total limb volume). The extremity volume was also correlated with the patient’s body weight, body mass index (BMI) and ideal body weight (IBW). The no-fat CT limb volume was correlated with the circumference-measured limb volume corrected by the ideal-body-weight to actual-body-weight ratio (IBW corrected-limb-volume).

**Results:**

The correlation between the CT volume and the volume measured by the circumference was high and significant. There was no correlation between the limb volume and the bare body weight, BMI or IBW. The correlation between the no-fat CT volume and IBW-corrected limb volume was high and significant.

**Conclusions:**

An appropriate drug dosing in ILP can be achieved by combining the limb volume with the simple circumference measurements and the IBW to body-weight ratio.

## Background

In light of the increasing incidence of obesity, there is an ongoing debate about the correct dosing of systemic chemotherapy and alternative weight models for the calculation of drug dosage [1,2]. For the treatment of locally advanced soft tissue sarcoma and malignant melanoma in the extremities, isolated limb perfusion with TNF-alpha and melphalan has proven to be one of the most effective treatment modalities with limb salvage rates of approximately 80% for soft tissue sarcoma [3-8] and local response rates of 90% for malignant melanoma [9-11].

Similar to systemic chemotherapy, the correct drug dosing is of great importance for isolated limb perfusion and isolated limb infusion, especially with TNF-alpha and melphalan (TM-ILP), to ensure the ideal balance of the maximum therapeutic effects with the lowest regional toxicity [12-15].

The majority of authors recommend determining the drug dosage according to the limb volume as first proposed by Wieberdink *et al*. [16,17]. There are some alternative dosage models that have been developed, such as dosage by body weight [18] and dosage by ideal body weight [19-21].

Though many recent articles do not explicitly mention the underlying drug dosage model, dosage according to the limb volume seems to be the leading dosage model. Measurements for the limb volume are acquired either by the original water displacement method described by Wieberdink or by circumference measurements, which are still performed in many European ILP centers, including our own [14,19].

Another alternative for exact volumetry is computed tomography (CT) scanning. CT scanning is most often used in liver transplantation and offers fast and accurate measurement of the organ volume [22-25]. Several authors have also described the use of CT scanning for volumetry in isolated limb perfusion (ILP)/isolated limb infusion (ILI), but due to the high cost and the radiation exposure, CT scanning for volumetry has not gained wide acceptance, despite its high accuracy.

Due to the high incidence of compartment syndrome at our institution between January 2011 and July 2011, we started investigating all possible factors that could influence compartment syndrome, including our drug dosage model. Since we do know from Deroose and coauthors that “dose matters” [12], we have not just chosen dose reduction to lower the risk for compartment syndrome because that would increase the risk for a reduced response of the tumor to the treatment.

Since the drug dose for melphalan was predefined (10 to 11 mg per liters of leg volume and 13 mg per liter of arm volume) [26,27] and only depended on the limb volume, we started our investigation by taking a closer look at the volume model used for calculating the absolute drug dosage because we believe that the exact dosage of the drugs is essential for maintaining the balance between a good response of the tumor and a low rate of local complications.

Considering the increasing interest in ILP and ILI and the number of newly developing ILP and ILI programs, we felt it was important to characterize the different drug dosage models and to develop a simple tool for estimating the limb volume and drug dosage in isolated limb perfusion.

The aim of this study was to validate a mathematical volume model for estimating the limb volume with simple circumference measurements and to compare the volume model data to weight-based drug dosage data, such as the actual body weight (ABW), body mass index (BMI) and ideal body weight (IBW). An additional aim of this study was to provide a web-based calculation tool that would allow for on- and offline calculation of standardized limb volumes [28].

## Methods

We performed a retrospective analysis of our ILP database and identified all patients who received an isolated limb perfusion and a digitally archived whole body staging positron emission tomography/computed tomography (PET/CT) that included the extremity before the ILP. All patients scheduled for ILP received a pre-operative limb volume estimation according to the circumference measure. Those patients who had evaluable CT data and a completely documented limb volume estimation (by the circumference measure) were included in this study.

### Calculation of limb volume by circumference measure

**Figure 1:**
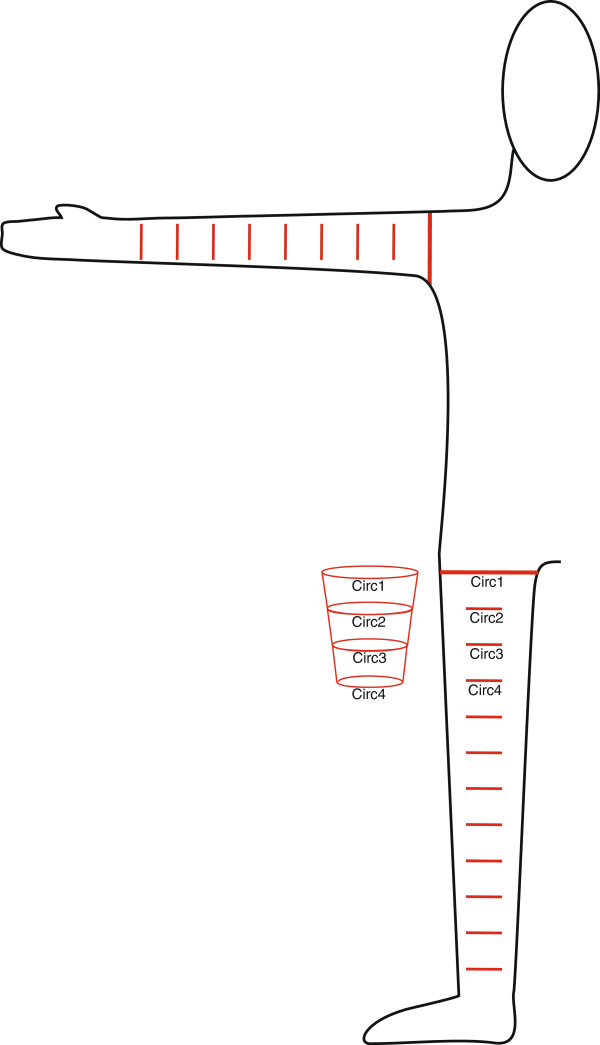
**Calculation of limb volume by circumference measure.** Measures are taken every 5 cm, and the volume of a truncated cone is calculated from two neighboring circumference measures. Volumes are summed, representing the total calculated limb volume.

The limb volume for the estimation of drug dosage was calculated by a standardized circumference measurement one day before the scheduled ILP. All measurements were performed while the patient was lying in a supine position. The patients’ legs were kept straight, and the arms were abducted to 90° at the shoulder. The extremity was marked with a waterproof pen; beginning at the groin/axilla, a base line was drawn (Figure 1), and markings were made at intervals of 5 cm down to the ankle-/wrist-level. In cases where the tumor affected the hand or foot, the distal extremity was included in the measurement. Beginning at the most proximal level, the circumference was measured at every 5 cm marking. All of the measured values were entered into an Excel-table (Microsoft Excel 2003, Microsoft, Redmond, WA, USA) or html/java-script-table [28].

The underlying calculation model was derived from a row of the frustums of a cone (Figure 1) that closely models the near circular volume of an extremity. The volume (V) of the frustum of a cone is calculated by $V=\frac{h\cdot \pi }{3}\cdot \left({\left(\frac{\text{Circ}1}{2\pi }\right)}^{2}+\left(\frac{\text{Circ}1}{2\pi }\right)\cdot \left(\frac{\text{Circ}2}{2\pi }\right)+{\left(\frac{\text{Circ}2}{2\pi }\right)}^{2}\right)$ with *h* being 5 cm (the distance for each measure), Circ1 and Circ2 being the two neighboring circumference measures. Each volume corresponds to that of a 5-cm segment of the extremity. The sum of all volumes equals the total volume of the extremity.

A copy of the html/java-script table is available for online use. The table can also be downloaded and stored for offline use on any java-script-enabled browser [28].

### Estimation of limb volume by CT

The reference limb volume for each patient was derived from CT data. Analysis was performed on the CT workstation (Syngo, Siemens AG, Berlin, Germany). As before, the proximal starting reference level was the axilla/groin, and the measurement was carried down to the wrist/ankle. As before, the distal extremity was included in the measurement in cases in which the tumor location was in the hand or foot. The extremity volume was derived from automatic data segmentation based on Hounsfield units (HU); the threshold was 200 HU, meaning that the surrounding air was not included, and everything above the density of 200 HU was included. The result was checked visually, and segmentation errors were corrected manually.

Second, the “no-fat limb volume”, representing the limb volume excluding the subcutaneous tissue, was measured as described above with the threshold set to -20 HU.

### Correlation between CT-calculated limb volume and circumference-measured limb volume

All calculations were performed in SPSS 20 (IBM Corporation, Armonk, NY, USA). The CT-calculated volume was correlated with the circumference-measured limb volume. The Pearson’s product-moment correlation coefficient (R) and its two-tailed significance were calculated. A normal distribution was tested by the one-sample Kolmogorov-Smirnov test.

### Correlation of limb volume with body weight, BMI and IBW

The circumference-measured limb volume was correlated with the patient’s ABW, BMI and IBW. The body mass index was calculated according to the World Health Organization (WHO) definition:

$$\text{BMI}=\text{Weight}\left(\text{kg}\right)/\text{Height}{\left(m\right)}^{2}.$$

The ideal body weight (IBW) was calculated by:

$$\begin{array}{ll} \;\text{IBW}\left(\text{kg}\right) & =49.9+0.89\\ & \;*\left(\text{Height}\left(m\right)–152.4\right)\;\text{for}\;\text{men}\;\text{and}\;\text{IBW}\left(\text{kg}\right)\\ & =45.4+0.89\\ & \;*\left(\text{Height}\left(m\right)–152.4\right)\;\text{for}\;\text{women}. \end{array}$$

Calculations were performed in separate groups for the arms and legs. Again, the Pearson’s R and its two-tailed significance were calculated.

### Correlation between limb volume corrected by ideal body weight and “no-fat CT volume”

To overcome the dilemma of the circumference-measured limb volume matching the true limb volume while lacking the possibility of fat reduction in obese patients, we adapted a model presented by Beasley and coauthors in 2008 to fit our volume model [29]. With this adjustment, we multiplied the measured limb volume with the quotient of the ideal body weight and the actual body weight.

$$V_{\text{corrected}}=V_{\text{measured}}\cdot \frac{\text{IBW}}{\text{ABW}}$$

This calculation results in a new parameter, the IBW-corrected limb volume, which represents the limb volume minus any excess fat-tissue caused by obesity. This parameter was correlated with the “no-fat CT limb volume” using the Pearson’s R and its two-tailed significance.

## Results

### Patient data

From our database, we identified 42 patients who received an isolated limb perfusion and underwent a pre-operative CT scan including the extremity. Of the 42 patients, 23 were male and 19 were female. The patients’ mean age was 56.4 (20 to 81) years. Thirty-two patients received a perfusion of the leg (12 iliac and 20 femoral) and 10 patients had perfusion of the arm (5 on the brachial level and 5 on the axillary level). Thirty-three (79%) ILPs were performed due to soft tissue sarcoma (STS), 5 due to melanoma (12%) and 4 (10%) due to other tumors, including high-grade chondrosarcoma, desmoid tumor, squamous cell carcinoma of the bone and lymphoma.

### Correlation between circumference measure and CT measure

**Figure 2:**
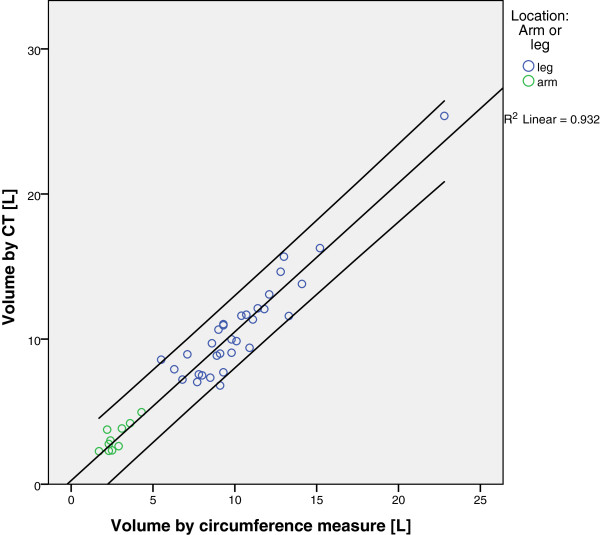
**Correlation of limb volume measured by circumference measure and limb volume measured by CT scan.** There is a very high linear correlation between the two measures. Green dots represent arms and blue dots represent legs. Note that the single dot to the far right represents a severely overweight patient who is still within the very narrow 95% confidence interval.

The mean volume estimated by CT was 10.8 (±3.673) liters for legs and 3.21 (±0.932) liters for arms. In both cases, the volume measured according to the circumference was slightly lower at 10.3 (±3.219) liters for legs and 2.73 (±0.765) liters for arms. Figure 2 shows the correlation between the circumference-measured volume and the CT-measured volume. Arms and legs had a nearly perfect match with the regression line; therefore, the mathematical correlation was performed for the arms and legs in a single group. The Pearson’s R was extremely high and significant (r = 0.965; *P* <0.01).

### Correlation of body weight, body mass index and ideal body weight

**Figure 3:**
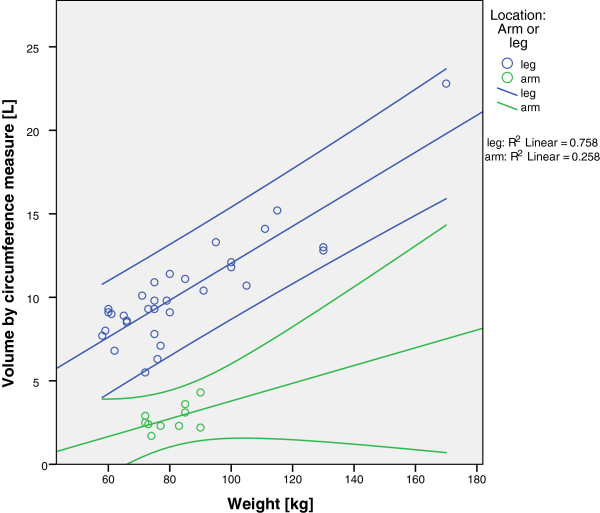
**Correlation of body weight and limb volume by circumference measure.** Green dots represent arms and blue dots represent legs. Notice that the 95% confidence intervals span a wide range.

**Figure 4:**
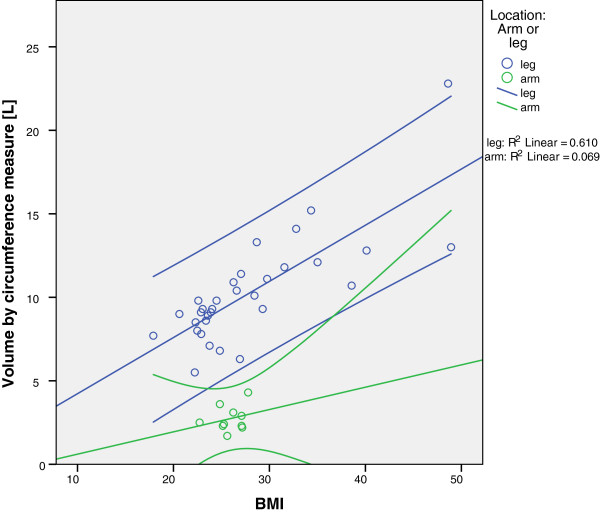
**Correlation between BMI and limb volume by circumference measure.** Green dots represent arms and blue dots represent legs. Again, the 95% confidence intervals span a wide range.

**Figure 5:**
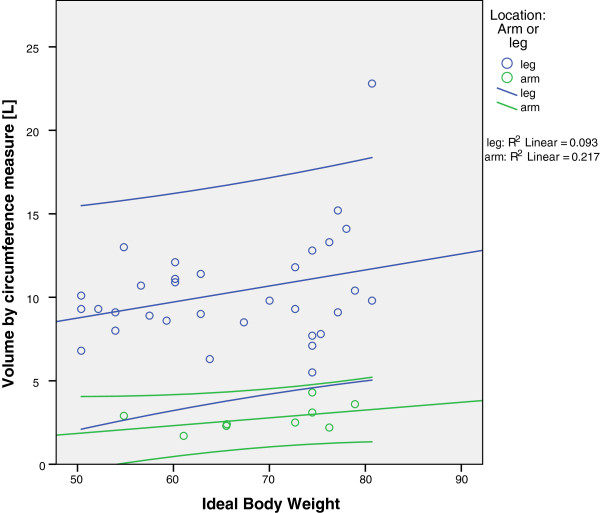
**Correlation of IBW and limb volume by circumference measure.** Green dots represent arms and blue dots represent legs. The coefficient of determination (R^2^) lies below 25% for both arms and legs, indicating that the IBW is the worst predictor for the extremity volume.

For all diagrams (Figures 3, 4 and 5), arms and legs were calculated separately. Figure 3 shows the correlation of the ABW with the limb volume (by the circumference measure). For legs, the correlation was positive and highly significant (r = 0.871; *P* <0.01); for arms, the correlation was not statistically significant (r = 0.508).

The correlation of the BMI and limb volume reveals a significant coherence for legs, as shown in Figure 4 (r = 0.781; *P* <0.01), while the correlation was not significant for arms (r = 0.263).

Not surprisingly, we found that the ideal body weight was the poorest predictor of the limb volume compared to the actual body weight and body mass index (Figure 5). In both cases (arm and legs), the correlation between the ideal body weight and the limb volume was not significant (r = 0.305 for legs; r = 0.466 for arms), which could be due to the inability of the ideal body weight to account for the excess fat tissue in obese patients.

### Correction between limb volume and ideal body weight

To overcome the dilemma of the circumference-measured limb volume matching the true limb volume while lacking the possibility of fat reduction in obese patients, the circumference-measured limb volume was corrected by the quotient of the IBW per ABW. The mean leg volume estimated by “no-fat CT” was 5.9 (±1.78) liters; the mean leg volume by IBW-corrected limb volume was 8.1 (±1.56) liters. Thus, the CT mean volume is 28% lower than the clinical volume. For arms, the “no-fat CT volume” was 1.8 (±0.75) liters and the IBW-corrected limb volume was 2.4 (±0.69) liters; thus, there was a 23% lower volume for the no-fat CT measure, which is expected because the “no-fat” CT scan subtracts all subcutaneous fat-tissue (in addition to the regular, non-obese fat).

**Figure 6:**
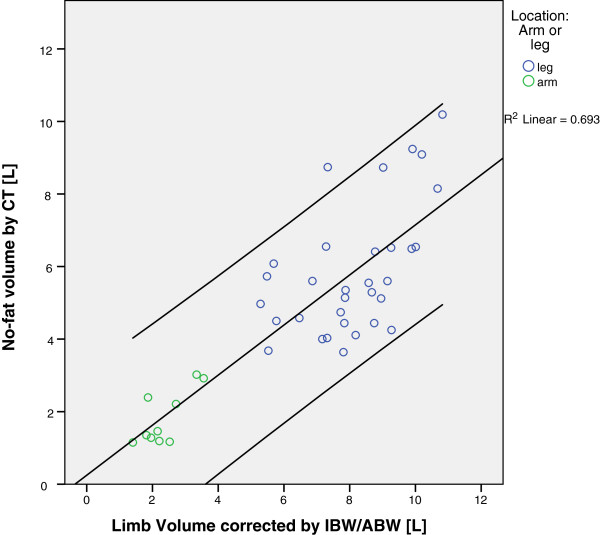
**Correlation between circumference measured limb volume corrected with IBW/ABW-ratio and “no-fat CT volume”.** The coefficient of determination (R^2^) shows that approximately 70% of all values fit the model.

As we can see in Figure 6, the correlation between the “no-fat CT volume” and the IBW-corrected limb volume is very high. The Pearson’s correlation coefficient was 0.833 and highly significant (*P* <0.01) for both arms and legs together. As shown above for the CT volume compared to the circumference-measured volume, the arm volume was lower -represented by the green dots - but matches the regression line like the legs (represented by the blue dots).

## Discussion

The safety of isolated limb perfusion and isolated limb infusion depends on a variety of parameters. One of these parameters is the correct dosage of the drugs administered during the procedure [13,30]. This is very much dependent on the volume of the extremity [16,17]. Arms and legs have very different limb volumes and factors such as age (especially in younger children), body weight and constitution of the patient that will ultimately affect the limb volume and recommended drug dosage. Thus, the exact dosage of the drugs is dependent on a precise volumetry of the limb in order to maintain the balance between a good response of the tumor and a low rate of local complications.

This study had the following three major aims: i.) to validate a circumference-measure volume model that was developed to allow for exact calculation of the limb volume for drug dosage in ILP; ii.) to compare body weight- and height-dependent measures to the limb volume model in terms of the accuracy of the model because the use of weight-based parameters for the dosage of drug calculation in ILP and ILI has recently become more popular [19,20]; and iii.) to develop a calculation tool that allows for standardized and safe volume measures and dosing of drugs in ILP and ILI.

We have shown that limb volume estimation by the circumference measure is highly reliable when compared to CT volume measurements. Even in obese patients, there is no relevant deviation in the circumference-measure volume model. In general, there is a smaller than 5% difference in the volume obtained with the circumference model and the corresponding CT-measured values. In contrast to the gold standard, the immersion method, where the extremity is immersed into a water container and the change of water level represents the exact limb volume as initially described by Wieberdink [16] has some major advantages. This method can be used for any patient, even highly immobilized or bedridden patients as Byrne and his co-authors have already described [18]. The method is cheap and easy to apply; it requires no special equipment apart from ordinary measuring tape and a personal computer with a java-script-enabled Internet browser.

Apart from the aforementioned points, there is one further advantage of note. If used with a waterproof marker pen, the circumference-measure method allows for easy dose adjustment in the femoral and brachial tourniquet placement. Because all frustums of a cone in the model are added successively, single volumes can be subtracted from the total volume, according to the true tourniquet placement in reference to the measurement markings on the patient’s skin. Especially in the leg, the proximal parts can easily make up half of the limb volume, which could lead to a drastic overdose if not subtracted.

An even easier alternative to the circumference volume measures is determining the limb volume or dose calculation by body weight- or height-dependent parameters [18-20], which has been described for systemic chemotherapy [1,2]. When using the ideal body weight as a parameter, a possible advantage is the fact that a high amount of fat tissue (especially in obese patients) will not be excessively represented in the limb volume [20,30].

As we have shown in this study, any body weight parameter by itself has a higher level of error compared to the limb volume. Especially for the arm, the deviation can easily reach 50% for patients with the same weight, BMI or IBW. In addition, as already mentioned, for femoral and brachial perfusions where the tourniquet can be placed way below the axillary or the groin level (depending on the location of the tumor), the limb volume is not actually perfused but is instead calculated by the weight, BMI or IBW models. This could account for a reduction in the perfused volume of up to 50%, which would lead to a drastic overdose.

The limb volume measurement is the most reliable parameter for the perfused volume compared to the CT measures, but it lacks a sufficient method of accounting for the excess body fat in obese patients. Beasley *et al*. have suggested a simple way of achieving the best of both worlds; the limb volume calculated by the circumference measure can be corrected by the quotient of the ideal body weight and actual body weight [29]. For a normal weight patient, the quotient will be close to 1, which means that the corrected volume will be equal to the actual limb volume. On the other hand, a severely obese (class III) patient will have a quotient of 0.5 or even less, which will lead to a 50% reduction of the limb volume, which is similar to what we have observed in the past by rough estimation.

In this study, we found that the limb volume measurement corrected by ideal body weight is the most appropriate method of limb volume estimation. This model shows a strong correlation with the no-fat PET-CT measures, meaning that by applying this method we achieve the best of both worlds. The individual limb volume is measured (subtract non-perfused parts of the extremity while taking high volume tumors into account) in combination with the ability to subtract only excess fatty tissue in the case of obesity.

Based on the results of this study, we have started to adopt the IBW-corrected volume model in our clinical practice. Further study is required to prove that this volume model leads to a reduction in the morbidity associated with compartment syndrome during ILP.

## Conclusions

In summary, this study has proven that the limb volume estimation by the circumference measure is a highly accurate model of the true limb volume. The advantage of this model lies in how easy it is to subtract the limb volume that is not perfused from the calculation. Because the use of body weight dependent parameters, such as the body weight, BMI or IBW, alone is inaccurate for determining the limb volume, it is necessary to correct the limb volume with the quotient of the ideal body weight and the actual body weight. This model was transferred into an html-java-script table that is available for download and offline use [28].

## Abbreviations

ABW: actual body weight; BMI: body mass index; CT: computed tomography; IBW: ideal body weight; ILP: isolated limb perfusion; ILI: isolated limb infusion; PET/CT: positron emission tomography/computed tomography; TM-ILP: TNF/Melphalan based isolated limb perfusion (in this article used synonymously with ILP); WHO: World Health Organization.

## Competing interests

The authors declare that they have no competing interests.

## Authors’ contributions

LEP designed this study (together with GT), developed the volume model and wrote the article. TP and MH are responsible for the CT-based volumetry data and contributed this section of the article. LD performed the actual volume measurements, searched the files for the volume- and all patient-related data. FG helped design the study and is responsible for maintaining the ILP database and keeping it up to date. GT developed the ILP database, contributed a majority of the patients and designed this study (together with LEP). All authors read and approved the final manuscript.
